# Clinical Impact of Inherited and Acquired Genetic Variants in Mastocytosis

**DOI:** 10.3390/ijms22010411

**Published:** 2021-01-02

**Authors:** Boguslaw Nedoszytko, Michel Arock, Jonathan J. Lyons, Guillaume Bachelot, Lawrence B. Schwartz, Andreas Reiter, Mohamad Jawhar, Juliana Schwaab, Magdalena Lange, Georg Greiner, Gregor Hoermann, Marek Niedoszytko, Dean D. Metcalfe, Peter Valent

**Affiliations:** 1 Department of Dermatology, Allergology and Venereology, Medical University of Gdansk, 80-211 Gdansk, Poland; magdalena.lange@gumed.edu.pl; 2 Department of Hematology, APHP, Hôpital Pitié-Salpêtrière and Sorbonne University, 75013 Paris, France; michel.arock@aphp.fr (M.A.); guillaume.bachelot@hotmail.fr (G.B.); 3 Centre de Recherche des Cordeliers, INSERM, Sorbonne Université, Université de Paris, Cell Death and Drug Resistance in Hematological Disorders Team, 75006 Paris, France; 4 Laboratory of Allergic Diseases, National Institute of Allergy and Infectious Diseases, National Institutes of Health, Bethesda, MD 20892-188, USA; jonathan.lyons@nih.gov (J.J.L.); dmetcalfe@niaid.nih.gov (D.D.M.); 5 Department of Internal Medicine, Division of Rheumatology, Allergy & Immunology, Virginia Commonwealth University, Richmond, VA 23298, USA; lawrence.schwartz@vcuhealth.org; 6 University Hospital Mannheim, Heidelberg University, 68167 Mannheim, Germany; andreas.reiter@medma.uni-heidelberg.de (A.R.); mohamad.jawhar@medma.uni-heidelberg.de (M.J.); Juliana.Schwaab@medma.uni-heidelberg.de (J.S.); 7 Department of Laboratory Medicine, Medical University of Vienna, 1090 Vienna, Austria; georg.greiner@meduniwien.ac.at; 8 Ludwig Boltzmann Institute for Hematology and Oncology, Medical University of Vienna, 1090 Vienna, Austria; gregor.hoermann@meduniwien.ac.at (G.H.); peter.valent@meduniwien.ac.at (P.V.); 9 Ihr Labor, Medical Diagnostic Laboratories, 1220 Vienna, Austria; 10 MLL Munich Leukemia Laboratory, 81377 Munich, Germany; 11 Department of Allergology, Medical University of Gdansk, 80-211 Gdansk, Poland; mnied@gumed.edu.pl; 12 Department of Internal Medicine I, Division of Hematology and Hemostaseology, Medical University of Vienna, 1090 Vienna, Austria

**Keywords:** mast cells, mast cell activation syndrome, gene polymorphisms, prognostication, hereditary alpha-tryptasemia, *KIT* variants

## Abstract

Mastocytosis is a rare and complex disease characterized by expansion of clonal mast cells (MC) in skin and/or various internal organ systems. Involvement of internal organs leads to the diagnosis of systemic mastocytosis (SM). The WHO classification divides SM into indolent SM, smoldering SM and advanced SM variants, including SM with an associated hematologic neoplasm, aggressive SM, and MC leukemia. Historically, genetic analysis of individuals with pure cutaneous mastocytosis (CM) and SM have focused primarily on cohort studies of inherited single nucleotide variants and acquired pathogenic variants. The most prevalent pathogenic variant (mutation) in patients with SM is *KIT* p.D816V, which is detectable in most adult patients. Other somatic mutations have also been identified—especially in advanced SM—in *TET2*, *SRSF2*, *ASXL1*, *RUNX1*, *CBL* and *JAK2*, and shown to impact clinical and cellular phenotypes. Although only small patient cohorts have been analyzed, disease associations have also been identified in several germline variants within genes encoding certain cytokines or their receptors (*IL13*, *IL6*, *IL6R*, *IL31*, *IL4R*) and toll-like receptors. More recently, an increased prevalence of hereditary alpha-tryptasemia (HαT) caused by increased *TPSAB1* copy number encoding alpha-tryptase has been described in patients with SM. Whereas HαT is found in 3–6% of general Western populations, it is identified in up to 17% of patients with SM. In the current manuscript we review the prevalence, functional role and clinical impact of various germline and somatic genetic variants in patients with mastocytosis.

## 1. Introduction 

Mastocytosis is a term used to define a group of rare myeloid neoplasms characterized by the expansion and accumulation of clonal (neoplastic) mast cells (MC) in the skin and/or in various internal organs. In common with normal MC, neoplastic MC are considered to derived from CD34+/CD38− stem cells [1,2]. However, in contrast to normal CD34+/CD38− stem cells, neoplastic stem cells in mastocytosis are transformed cells that undergo clonal evolution and sub-clone formation depending on the acquisition of somatic mutations in critical target genes [3,4,5].

In general, cutaneous forms of mastocytosis (CM) and variants of systemic mastocytosis (SM) have been described [6,7,8,9,10,11]. The World Health Organization (WHO) divides SM into indolent SM (ISM), smoldering SM (SSM), SM with an associated hematologic neoplasm (SM-AHN), aggressive SM (ASM), and MC leukemia (MCL)—Table 1 [9,10,11].

The diagnosis of SM is based on WHO criteria, including abnormal histopathological and cytological aspects of MC in the bone marrow (BM) or in other internal organs, an elevated basal serum tryptase (BST) level, an aberrant immunophenotype of MC (CD2^+^/CD25^+^), and/or identification of the somatic mutation (pathogenic variant) *KIT* p.D816V [9,10,11]. Most adult patients with SM presenting with ISM (>80% cases) and have a normal or near-normal life expectancy [6,7,8,9,10,11]. Only a few of these ISM patients (<5%) progress to advanced SM [9,10,11]. Patients with advanced SM, including SM-AHN, ASM and MCL have a poor prognosis with reduced overall and progression-free survival [9,10,11,12,13]. In these patients, MC infiltration leads to organ damage [9,10,11,12,13]. In many cases, the disease is resistant to most conventional and targeted drugs including tyrosine kinase inhibitors (TKI).

From a molecular point of view, SM can be divided into three groups: (i) SM with the *KIT* p.D816V variant detected only in MC and a few other cells, (ii) multi-lineage SM where *KIT* p.D816V is also detected in most or all other myeloid cells in the BM and blood, (iii) and multi-mutated SM where, in addition to *KIT* p.D816V, mutations in other myeloid malignancy-related genes, such as *TET2* or *ASXL1* are found [14,15,16,17,18].

The diagnosis of CM is based on typical cutaneous features, including typical macular or maculopapular skin lesions and a positive Darier’s sign, as well as an increase in MC in lesional skin. The WHO recognizes 3 forms of cutaneous mastocytosis (CM): maculopapular CM (MPCM), diffuse CM, and mastocytoma of skin [9,10,11,19]. The majority of CM patients are children. The prognosis in both children and adults with CM is excellent. In these patients, transition to SM may occur but is rare and progression of true CM into advanced SM is very unusual [19,20,21,22].

MC activation syndrome (MCAS) is a condition defined by severe systemic symptoms induced by MC-derived mediators. MCAS can be primary, such as in SM or clonal MCAS, secondary, or idiopathic. Most MCAS cases present with clinical signs of anaphylaxis which in the case of secondary MCAS may result from an underlying IgE-dependent allergy [23,24,25].

## 2. Studies of the Common Germline Genetic Variants in Patients with Mastocytosis 

The presence of activating *KIT* mutations alone is not sufficient to explain the different clinical forms of mastocytosis, suggesting that other inherited or somatic genetic variants are important in the regulation of MC proliferation and/or activation.

Genetic studies in CM and SM have focused on genes encoding cytokines and their receptors (*IL13*, *IL6*, *IL6R*, *IL31*, *IL4R*) or on genes encoding Toll-like receptors (TLRs) [26,27,28,29,30].However, given the rarity of mastocytosis only small numbers of patients have been studied and more studies are required to draw definitive conclusions regarding the impact of these genetic variants on phenotypes and outcomes associated with mastocytosis.

### 2.1. IL4R and IL13 Variants

Interleukin 13 and 4 are pleiotropic cytokines that are 20–25% identical and have similar effector functions within the type 2 immune response [31,32]. Both IL-4 and IL-13 are capable of signaling through the shared type-II IL-4 receptor, which is a heterodimer consisting of IL-4 receptor α (IL-4Rα) encoded by *IL4R*, and IL-13 receptor α 1 (IL-13Rα1) [33,34]. In the context of MC, IL-13 has been shown to induce expression of SCF, IL-6, and MCP-1 (monocyte chemotactic protein-1) [35,36]. Results of linkage and GWAS studies indicated that polymorphisms of *IL4*, *IL4R* and *IL13* genes could be associated with total serum IgE levels in patients with allergies such as asthma and atopic dermatitis [37]

One study examining the role of genetic variants in this pathway was conducted by Daley, Metcalfe and Akin. They identified an association between the gain-of-function variant p.Q576R (rs 1801275) in the *IL4R* gene and CM, wherein the presence of this missense variant was associated with a more favorable prognosis [26]—Table 2. The authors found that the presence of the *IL4R* p.Q576R missense was also associated with a lower MC burden as determined both by the extent of clinical disease and by circulating levels of surrogate disease markers (i.e., tryptase and soluble CD117) and suggested that increased IL-4 and/or IL-13 signaling may limit tissue MC numbers in patients with mastocytosis [26].

In contrast to these findings, suggesting that increased IL-4 and/or IL-13 signaling may limit MC numbers and disease severity in patients with CM, Nedoszytko et al. [27] identified a distinct pattern associated with IL-13 in patients with mastocytosis—Table 2. In fact, when examining the promoter polymorphism −1112C > T (rs 1800925) in the *IL13* gene in CM and SM patients [21], it appeared that the high transcription variant (−1112T) may be present more frequently in mastocytosis patients than in controls, and was significantly more common among patients with SM as compared to those with CM [27]. The −1112C > T polymorphism was also found to correlate with an elevated serum tryptase level and with adult-onset of the disease, both of which are almost invariably associated with SM. Examined patients with SM also exhibited elevated serum IL-13 levels [21]. Moreover, the human MC line HMC-1 was found to display a functional IL-13 receptor [27]. Together, these data suggest that the *IL13* high transcription promoter variant and elevated IL-13 levels may contribute to MC expansion and may thus promote evolution of SM [27].

However, in all these studies only a limited number of patients with SM or CM have been examined. Therefore, it remains unknown whether these cytokine and cytokine receptor variants are indeed relevant clinically. Future studies with more patients are required to draw definitive conclusions on the role of altered signaling in the IL-4 and IL-13 signaling pathways in CM and SM variants.

### 2.2. IL31 Variants

Interleukin 31 is a member of the IL-6 cytokine superfamily and is secreted by a variety of cells including activated type 2 CD4^+^ T cells and MCs [38,39,40,41]. Interleukin 31 has been reported to play a role in the etiology of pruritus, chronic inflammation and regulation of innate and adaptive immunity in tissues exposed to environmental factors. Interleukin 31 has also been demonstrated to be involved in the pathogenesis of chronic skin inflammation and pruritus in patients with chronic spontaneous urticaria, atopic dermatitis, prurigo nodularis, primary cutaneous lymphomas and myeloproliferative disorders including mastocytosis [39,40,41,42,43,44].

Several variants of the *IL31* gene have been reported in association with atopic dermatitis [45,46,47]. Recently, Hartmann et al. [44] have shown that patients with mastocytosis have increased serum IL-31 levels and that serum IL-31 levels correlate with the disease severity, tryptase levels, and the degree of BM MC infiltration in adult patients with mastocytosis. A study by Lange et al. confirmed increased serum levels of IL-31 in mastocytosis patients and reported that IL-31 levels in pruritic cases of mastocytosis were significantly higher than in non-pruritic cases [28]. Authors analyzed association of mastocytosis with two variants in promoter *IL31* gene (−1066 G > A (rs 11608363), −2057 G > A (rs 6489188), and one in intronic region (IVS2 + 12A > G) and found that −2057AA genotype is associated with a high prevalence of mastocytosis (CM and SM) in adult patients. They also identified an association between non-coding *IL31* variants and an increased risk for CM in adults and children (Table 3).

### 2.3. IL6 and IL6R Variants

Interleukin 6 (IL-6) is a multifunctional inflammatory cytokine that plays a role in the regulation of cell proliferation, hematopoiesis, immune response, and inflammation. Interleukin 6 is a major MC-derived mediator and is elevated in patients with mastocytosis [48,49,50,51,52,53,54].

Interleukin 6 may also act as an autocrine growth factor for neoplastic MC [53,54]. Brockow et al. demonstrated that IL-6 plasma levels, but not sIL-6R levels, were elevated in patients with mastocytosis and correlated with BM pathology, organomegaly, and the extent of skin involvement. In plasma, there was a positive correlation of IL-6 levels with total tryptase levels, alkaline phosphatase, IgM, and white blood cell counts [48,49]. In addition, increased IL-6 serum levels correlated with severity of symptoms, the SCORMA (SCORing MAstocytosis) index, disease progression, and the presence of osteoporosis [49,51,53].

An examination of genetic variants in this pathway was undertaken by Rausz et al. [29] where the frequency of the −174G > C (rs 1800795) variant in the *IL6* promoter and prevalence of the common p.D358A missense variant in *IL6R* were determined in mastocytosis patients. The authors showed that homozygous carriers of the missense variant of the *IL6R* gene (rs 9192284 AA genotype) had a 2.5-fold lower risk for mastocytosis than those with the AC or CC genotypes. No association with mastocytosis was found for the *IL6* promoter variant—Table 4 [29].

### 2.4. TLR2 Gene Variants

Toll-like receptors (TLRs) are essential members of the pathogen recognition receptor family, expressed broadly by immune and non-immune cells including MC. All but two of the ten known TLR—the exceptions being TLR-8 and -9—have been reported to be expressed on human MC [55]. TLR-1/2, TLR-2/6, TLR-4, and TLR-5 receptors activate P13K-AKT, MAPK, and increase production of eicosanoid and pro-inflammatory cytokines, including TNF-α, IL-1β, and IL-6 [55,56,57]. Several variants in *TLR* genes that affect these signaling pathways have been described [52,53,54]. In certain infectious, inflammatory, and allergic diseases, the missense variants *TLR2* p.R753Q and *TLR4* p.D299E, and the *TLR9* promoter variant −1237C > T have been associated with increased signaling [58,59,60,61].

In 2018, Nedoszytko et al. examined the frequency of the *TLR2* p.R753Q (rs5743708) variant, an intronic *TLR4* variant 896A > G (rs496790), and the −1237C > T (rs5743836) *TLR9* promotor variant in CM and SM patients—Table 5 [30]. In this study, the *TLR2* p.R753Q variant was significantly more common among patients with mastocytosis compared to healthy controls and in particular those with systemic disease compared to controls. The presence of the *TLR2* p.R753Q variant was also associated with a 2-fold increased risk for mastocytosis, and 4-fold risk for systemic disease. In contrast, the *TLR4* and *TLR9* variants examined were not associated with disease [30].

## 3. mRNA Expression Studies in Patients with Mastocytosis

While few studies published to date have examined differences in gene expression patterns in bone marrow (BM) and peripheral blood cells from mastocytosis patients, a study by d’Ambrosio et al. [62] first suggested that mastocytosis patients have altered RNA expression profiles in BM cells. The most over-expressed gene products included genes encoding secreted tryptases, carboxypeptidase A, and proteins involved in regulation of transcription, proliferation, and apoptosis. The expression level of 3 genes (*TPSAB1* encoding α- and β-tryptases, *ATF3* encoding Activating transcription factor type 3, and *MAFF* encoding the Muscle aponeurotic fibrosarcoma type F oncogene) were significantly associated with serum tryptase levels and these 3 genes have been suggested as candidate molecular markers for systemic disease.

In a second similar study, Teodosio et al. [63] analyzed the gene expression profile in highly purified BM MC from ISM and ASM mastocytosis patients with *KIT* p.D816V. In comparison to controls, 758 genes were deregulated in ISM/ASM patients. Patients with ISM had fewer deregulated genes (*n* = 479) than patients with ASM (*n* = 677); more than half (398/758) of these genes were shared by both groups of patients. The most deregulated genes were *KIT*, *CD25*, *TPSAB1*, and gene products involved in the innate and inflammatory immune response including IL-1 receptor type 1 (IL1R1), CCL23, and CD4, as well as interferon-induced gene products and gene products involved in cellular responses to viral antigens, together with complement inhibitory molecules and genes involved in lipid metabolism and protein processing.

Purified BM MC from patients with ISM have been found to have a number of genes upregulated that are classically associated with signaling pathways involved in MC degranulation, such as *Linker for activation of T cells*, *member 2* (*LAT2*) and *CD33* encoding Siglec-3, as well as significant enrichment in genes involved in cell adhesion including *Cadherin 12* (*CDH12*) and *CD81* encoding tetraspanin-28. By contrast, purified BM MCs from patients with ASM displayed upregulation of genes principally related to inflammation and apoptosis, including *Caspase 1* (*CASP1*), *Caspase 10* (*CASP10*), *XIAP associated factor 1* (*XAF1*), *Tumor necrosis factor receptor superfamily member 10B* (*TNFRSF10B*), *Interleukin 1 beta* (*IL-1B*), and *Oncostatin M* (*OSM*) [63].

More recently, Niedoszytko et al. [64] have compared global gene expression in peripheral blood cells obtained from ISM patients to those from healthy controls. A total of 2330 mRNA transcripts were found to be differentially expressed in leukocytes from ISM patients compared to controls. Of these, 1951 gene products (84%) were upregulated in ISM patients and 379 (16%) were downregulated. The main pathways in which the differentially expressed mRNA were identified included ubiquitin-mediated proteolysis, as well as JAK-STAT, MAPK, and p53 signaling pathways. In another study significant differences were reported among ISM patient with insect venom anaphylaxis as compared to those without anaphylaxis in history [65].

Finally, in a study by Górska et al. [66] of gene expression in mastocytosis patients, increased expression of *TRAF4* mRNA was associated with food hypersensitivity and decreased expression of *B3GAT1* mRNA was associated with insect venom allergy.

In all these studies, the numbers of patients were limited. Therefore, the clinical impact of these gene products and their specific influence on clinical variables, presentations and courses remains uncertain. In addition, all these studies were retrospective in nature. Therefore, prospective studies including higher numbers of patients are required to draw definitive conclusions regarding the clinical and prognostic impact of these preliminary findings.

## 4. Missense *MRGPRX2*, *ADGRE2,* and *PLCG2* Genes Variants and Mast Cell Activation

Two primary pathways have been identified in MC activation: Immunoglobulin E (IgE)-dependent and IgE-independent pathways. In the IgE-independent pathway, MCs are activated by stimulation of various receptors: IL6R, IL18R, ST2, TLR, KIT, histamine receptors, IgGR, C3aR, C5aR, and the G protein-coupled receptors (GPCRs) ADREG2 and MRGPRX2, though many of these pathways are insufficient to result in MC degranulation [56,67,68,69,70,71,72,73,74,75,76,77,78].

MRGPRX2 (Mas-related G protein-coupled receptor) is activated by different ligands including but not limited to: substance P, MC degranulating peptide, neuropeptide Y, VIP, somatostatin, compound 48/80, fluoroquinolone antibiotics, as well as the antimicrobial peptides LL37 and HBD, and insect venom peptides melittin and vespid mastoparan [69,70,71,72]. Recently, gain- and loss-of-function variants in *MRGPRX2* were found to impact MC degranulation in vitro, in response to substance P and other peptides [73,74]. While it has been suggested that quinolones are potential triggers of anaphylaxis in mastocytosis [74,75], these variants have yet to be shown to impact clinical phenotypes in humans.

ADGRE2 (Adhesion G protein–coupled receptor E2) is a mechanoreceptor present on MC. The *ADGRE2* p.C492Y missense has been shown to result in a severe autosomal dominant form of vibratory urticaria. In patients with this variant, vibration-induced activation of MC results in diffuse tryptase staining in skin upon histologic staining, and the rapid increase in histamine levels in venous blood from challenged areas [76].

Another study by Ombrello et al. [77] showed that variants of *PLCG2* gene encoding Phospholipase Cγ_2_ (PLCγ_2_) are involved in the pathogenesis of cold-induced urticaria. Cold-induced urticaria is a unique inflammatory disorder that is characterized by MC degranulation and triggered by exposure to cold stimuli, a condition that can culminate in life-threatening anaphylaxis [77,78]. In three examined families with this disease the authors showed that deletions of auto-regulatory domains of *PLCG2* resulted in protein products with constitutive phospholipase activity under cold temperature conditions, while they remained hippomorphic at physiologic conditions [78]. This unusual dichotomy resulted in impaired humoral immunity and enhanced MC and granulocyte-mediated cutaneous inflammation [79].

Variants in genes such as *MRGPRX2*, *ADGRE2* and *PLCG2* have not been extensively examined among individuals with mastocytosis. However, we consider these molecules and their pathologic variants as candidates and promising targets mediating MC activation in patients with CM or SM. Whether indeed these molecules play a major role in MC activation or MCAS and whether indeed these targets can be exploited to establish new therapies against MC activation in mastocytosis contexts remains to be examined in forthcoming studies.

## 5. Hereditary Alpha-Tryptasemia in Clonal MC Disease

In 2014, it was first recognized by Lyons and colleagues that elevated basal serum tryptase (BST) levels could be inherited in humans in an autosomal dominant manner [80]. In that study, elevated BST was associated with multisystem complaints, including symptoms of MC mediator release such as abdominal pain, bloating, diarrhea, skin itching, flushing—and in some cases urticaria—as well as an increased prevalence of anaphylaxis in affected individuals. In a follow-up study in 2016 [81], the investigators went on to describe the basis for this finding, a common genetic trait they termed hereditary alpha-tryptasemia (HαT). HαT is caused by increased α-tryptase encoding *TPSAB1* copy number [82] and is a common genetic cause for elevated BST in Western populations, affecting between 4–6% of all individuals [81,83,84,85]. Among these individuals with HαT a gene dosage effect has also been observed, where increasing *TPSAB1* copy numbers are associated with higher BST levels and more prevalent clinical symptoms. Increasing ratios of α-tryptase to β-tryptase encoding genes also correspond to the portion of active tryptase in mast cells accounted for by α/β-tryptase heterotetramers; such heterotetramers (but not the homotetramers) are capable of activating protease-activated receptor-2 and ADGRE2, which in turn may contribute to aspects of anaphylactic severity [84,86].

In initial studies, 16% of affected individuals in families recruited with HαT reported systemic anaphylaxis to Hymenoptera envenomation (HVA). Moreover, in a small validation cohort of ostensibly healthy adult volunteers, the relative risk for HVA among individuals with HαT was found to be 9.1 [81]. In order to validate this association, two large cohorts of venom allergic patients from Italy and Slovenia were tryptase-genotyped, and in both cohorts, the prevalence of HαT among individuals with severe HVA (grade IV on the Mueller scale) was found to be at least twice that of the general population and that of those with less severe anaphylaxis (grades I–III) [84]. Importantly, the prevalence of HαT was not found to be increased among venom allergic patients *en masse*, indicating that HαT is not likely to affect sensitization or peripheral tolerance, rather, that among those who are allergic, reactions are more likely to be severe. Whether anaphylaxis resulting from other allergens is similarly modified by HαT in *healthy* individuals has not yet been determined. However, individuals with idiopathic anaphylaxis in the absence of clonal MC disease have been reported to be three times as likely to have HαT, suggesting that this may be a common phenomenon. Indeed, proposed mechanisms—involving specific cleavage and activation of EMR2 on MC cells and PAR2 on endothelial cells by mature α/β-tryptase heterotetramers—would appear to be generalizable [84,86].

The possibility that HαT might modify clonal MC disease was first suggested following the identification of a highly symptomatic family with HαT in whom the first *TPSAB1* quintuplication was found [87]. In this family, one affected individual also had evidence of clonal MC disease, harboring the activating *KIT* p.D816V missense. The authors had previously noted that HαT was associated with increased MC numbers in the BM of affected individuals [82] and surmised that since a *TPSAB1* quintuplication is a rare finding, also identifying similarly rare clonal MC disease in a single individual was unlikely to be happenstance. Thus, the investigators went on to examine the prevalence of HαT in clonal MC disease finding that 12.2% of individuals with SM followed at the U.S. National Institutes of Health (NIH) had concomitant HαT, a rate more than twice that of the general population [84]. In a follow-up study, performed in collaboration between the Gdansk and Vienna European Competence Network on Mastocytosis (ECNM) centers, this association was validated, and an even greater prevalence was seen [83]. A total of 17.2% of the 241 individuals with SM included in the study were found to have HαT. In both the U.S. and European studies, HαT appeared to modify the clinical course and phenotype associated with mastocytosis, making anaphylaxis more prevalent and more severe [83,84]. Moreover, in the latter study—much like what was reported in the initial description of HαT—investigators observed a gene dosage effect on clinical symptoms [83]. Using a validated mediator symptom severity grading system, Greiner and colleagues found that with increasing α-tryptase-encoding *TPSAB1* copy, more pervasive symptoms and higher mediator-associated symptom grades were reported by their patients [83].

Data remain limited about HαT-associated augmentation of MC mediator production, release, and/or responsiveness in patients with allergies and MC disorders. However, IgE-mediated degranulation of primary MCs cultured from individuals with HαT has not been shown to be different from controls [81]. Given the finding of increased bone marrow MC in the absence of clonal disease among individuals with HαT, one could consider whether HαT may facilitate or promote the evolution of this clonal myeloid disorder. However, because HαT significantly modifies clinical phenotypes associated with clonal MC disease, its increased prevalence among patients with SM may also reflect a detection bias, wherein those with both HαT and mastocytosis are more likely to come to medical attention because of increased symptom severity. As additional clinical, epidemiologic, and basic research is conducted on this common genetic trait, more insights will be gained into the role that HαT plays in MC expansion and reactivity in SM and atopic disorders.

## 6. *KIT* Variants and Their Role in the Pathogenesis of Mastocytosis 

KIT is activated by stem cell factor (SCF) and is a type III transmembrane receptor with intrinsic tyrosine kinase (TK) activity in its intracellular domain. KIT, which is expressed by germ cells, Cajal cells, melanoblasts, hematopoietic stem cells (HSC) and MC, is deeply involved in hematopoiesis and mastopoiesis. In particular, KIT activity plays a crucial role in the survival and proliferation of HSC and in the development of MC [88]. In the MC lineage, KIT is thus expressed throughout differentiation and appears to play a critical role in the differentiation, proliferation, survival and migration of MC and their progenitors [88].

*KIT* mutations are thought to be involved in the pathogenesis of the majority—if not all—variants of mastocytosis. Indeed, in CM as well as in SM, disease pathogenesis is considered to be associated with the presence of acquired gain-of-function variants in the *KIT* gene. Most of these mutations lead to constitutive activation of the KIT receptor and thus autonomous (SCF-independent) development and accumulation of MC [89]. The KIT variants detected in mastocytosis can be classified into two categories. The first category includes regulatory variants, affecting the dimerization or conformational self-regulation of the receptor. These variants are often found in pediatric mastocytosis and are typically located in the extracellular domain (ECD) encoded by exons 2, 8 and 9 of the KIT gene [90]. The second category of variants occurs at the intracellular portion of the receptor, in the second kinase domain (KD2) encoded by exon 17 of the KIT gene [14].

The *KIT* variant most frequently encountered in adults with SM (more than 80% of all SM cases and >90% of patients with ISM) is KIT p.D816V, which occurs in the KD2 of KIT [91]. Other *KIT* mutations are found less frequently in SM patients. Such alternative *KIT* mutations are particularly detected in patients with ASM, MCL, or MC sarcoma (MCS)—these variants are often located in the juxtamembrane domain (JMD), such as *KIT* p.V560G, or in the ECD of the receptor, such as *KIT* p.Del419 [92]. Finally, some patients with SM do not have identifiable variants in *KIT* (*KIT* wild type; *KIT* WT); this is particularly the case in the nearly 70% of patients who present with well-differentiated SM (WDSM), a subset of ISM characterized by the presence of compact multifocal infiltrates of round mature, CD2- and CD25-negative MC in BM [93].

**Figure 1:**
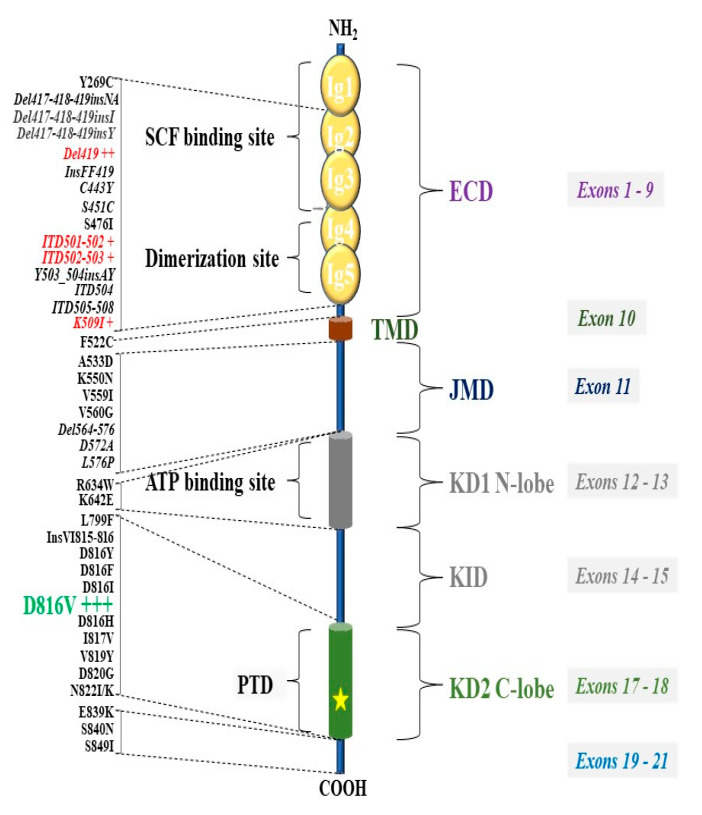
KIT variants reported in mastocytosis. Variants frequently observed in pediatric patients with CM are shown in red, whereas the KIT p.D816V mutation (pathogenic variant) recurrently found in adult SM patients is shown in green. Abbreviation: ECD: Extracellular domain; JMD: Juxtamembrane domain; KID: Kinase insert domain; PTD: phosphotransferase domain; KD1, tyrosine kinase domains; TMD: transmembrane domain.

**Figure 2:**
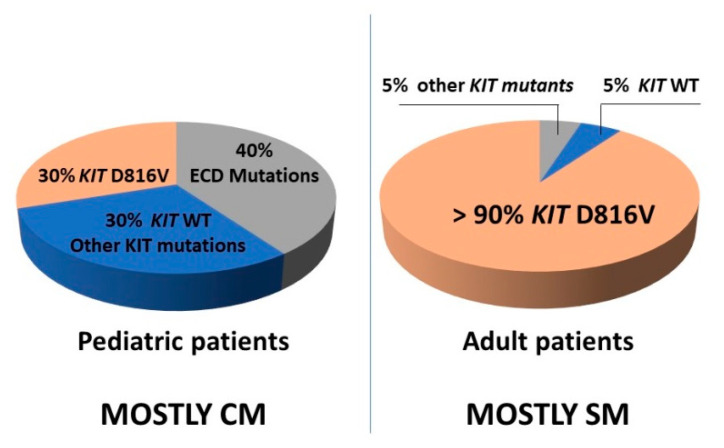
Frequency of *KIT* variants in pediatric and adult patients with mastocytosis. Abbreviation: CM: cutaneous mastocytosis; ECD: Extracellular domain; *KIT* WT: *KIT* wild-type; SM: systemic mastocytosis.

The *KIT* p.D816V mutation is found in only ~30% of children with CM, while other *KIT* variants, located mainly in the ECD of the receptor, are found in ~40% of these cases [90]. An updated list of *KIT* variants described in mastocytosis is provided in Figure 1, and a comparison of the frequency of different *KIT* variants found in adults vs children is shown in Figure 2.

In adult SM patients, *KIT* variants may be found in hematopoietic cells other than MCs, suggesting involvement of other cell lineages and their progenitors in these cases [94]. This condition is more frequently seen in patients with smoldering SM (SSM) and advanced SM, and is associated with a higher risk of disease progression [95]. In patients with SM-AHN, the *KIT* p.D816V variant is expressed not only in MC but can also be detected in other cell lineages affected by the AHN [96].

Even today, the mechanism(s) of the constitutive activation of the KIT mutant receptors is (are) not yet fully understood, particularly for *KIT* p.D816V. However, several hypotheses have been proposed, such as: (a) homologous or heterologous spontaneous dimerization of the receptor in the absence of SCF; (b) triggering of the signaling cascade by a single monomer or; (c) dysfunction of the JMD and of its self-inhibitory action [97].

Interestingly, there are several discrepancies between wild-type (WT) KIT and mutant KIT p.D816V receptor signaling, which suggest the existence of molecular differences specific to oncogenic signaling. For instance, following the activation of WT KIT, a number of negative regulatory mechanisms and pathways are canonically activated. These regulatory feed-back mechanisms involve diverse pathways, such as certain phosphatases, endocytosis-related mechanisms, and degradation of receptor molecules [98]. In mastocytosis, many of these regulatory systems have been shown to no longer control KIT signaling [98]. Mutated KIT receptor—which is constitutively active—is usually a cytoplasmic molecule and in contrast to WT KIT, is not expressed on the cell surface. The KIT p.D816V mutant receptor has the ability to escape inhibitory effects of phosphatases. Indeed, the phosphatase SHP-1 (Src Homology region domain-containing Phosphatase-1), involved in the negative regulation of KIT, seems to be degraded rapidly in a mouse cell model exhibiting the *Kit* p.D814Y variant (equivalent to the human *KIT* p.D816V mutation) [99].

With regard to the oncogenic pathway evoked by *KIT* p.D816V, two pathways seem to play a central role in the pathogenesis of clonal disease in patients with *KIT* p.D816V SM, i.e., the STAT5 and the PI3K/AKT pathways [100,101]. STAT5 is constitutively activated in neoplastic cells from patients with *KIT* p.D816V-positive SM, as reflected by the presence of phospho-STAT5 (pSTAT5) at the nuclear and cytoplasmic level. Of note, pSTAT5 is found bound to p85 (PI3K regulatory subunit) by Gab2 in the cytoplasm of neoplastic MC [101]. In vitro, in two human neoplastic MC lines harboring the *KIT* p.D816V variant, namely HMC-1.2 and ROSA^KIT D816V^, not only STAT5 but also AKT have been found to be constitutively activated [102,103].

In addition, other kinases appear involved in the oncogenic signaling resulting from the *KIT* p.D816V mutation. Indeed, the FES tyrosine kinase (“FEline Sarcoma”) seems to be a major effector of proliferation through its role as a regulator of STAT and mTOR signaling, whereas it is not involved in the proliferation mediated by WT KIT [104,105]. Its activity is increased in malignant MC and it phosphorylates ribosomal protein kinase S6 (P70S6K), a serine/threonine kinase which impacts several genes regulating cell growth, survival, and metabolism [106].

Apart from KIT p.D816V, neoplastic MC may express a number of other molecules believed to be involved in their abnormal expansion and accumulation. Indeed, neoplastic MC may also overexpress LYN and BTK, Oncostatin M (OSM), NF-κB, and several anti-apoptotic molecules, such as Microphtalmia-associated Transcription Factor (MITF) and the BCL2 Family Member MCL-1 [107,108,109]. Finally, it has been reported that in neoplastic MC the expression of pro-apoptotic molecules such as BIM are decreased [110]. All these events may contribute to the pathological accumulation of MC in mastocytosis.

## 7. Role of Additional Genetic Variants (Mutations) as Drivers of Advanced SM

KIT p.D816V is considered to be a critical driver of SM. However, the presence of this mutation alone does not explain the diverse clinical levels of aggressiveness of clinical variants of SM [95].

Recent studies have shown that in >60% of all patients with advanced SM (ASM, SM-AHN or MCL), neoplastic cells harbor somatic variants in relevant genes other than KIT [111,112,113,114,115,116,117]. These additional mutations affect genes encoding transcription factors (e.g., *RUNX1*), signaling molecules (*JAK*, *KRAS*, *NRAS*, *CBL*), epigenetic regulators (*TET2*, *ASXL1*, *DNMT3A*, *EZH2*) or splicing factors (*SRSF2*, *SF3B1*, *U2AF1*). In the study by Schwaab et al. [3], the most frequently affected genes were *TET2*, *SRSF2*, *ASXL1*, *CBL*, and *RUNX1*. In advanced SM, 21/27 patients (78%) carried ≥3 variants, and 11/27 patients (41%) exhibited ≥5 variants. Overall survival was significantly shorter in patients with additional aberrations as compared to those identified as having KIT p.D816V alone.

Several groups have investigated the prognostic relevance of additional variants in myeloid malignancy-related genes [111,112,113,114,115,116,117]. Jawhar et al. [111,112] have reported that variants in 3 critical genes, namely *SRSF2*, *ASXL1*, and *RUNX1* (*S/A/R* gene panel) can influence overall survival and are thus the most important predictive indicators of a poor outcome in patients with SM. Finally, the presence and number of mutated genes within the *S/A/R* panel are associated with the presence of advanced SM, especially SM-AHN [112,116].

Munoz-Gonzales et al. have since demonstrated that in addition to the *S/A/R* gene panel, somatic *EZH2* gene variants might also provide prognostic information and have proposed an S/A/R/E panel [116]. In the Mayo prognostic score, *ASXL1*, *RUNX1*, and *NRAS* gene variants were found to be independent predictors of an inferior survival in SM [117]. Moreover, additional variants in the genes *ASXL1*, *RUNX1*, and *DNMT3A* have also been described as prognostic factors in indolent SM [16].

In conclusion, the clinical and morphological diversity of SM is associated with the presence and number of somatic variants in certain target genes, and the respective gene products are considered to play a role in the pathogenesis, aggressiveness and poor prognosis of patients with advanced SM. For this reason, the current consensus is that molecular signatures should be determined in all patients with SM.

## 8. Conclusions

Molecular studies in patients with mastocytosis have demonstrated a multifactorial genetic basis with disease being dictated by somatic mutations in critical driver genes—including recurrent gain-of-function mutations in *KIT*—and expressivity being significantly modified by heritable genetic modifiers, including hereditary alpha-tryptasemia. In more than 80% of all patients with SM, neoplastic cells harbor a somatic mutation in *KIT* at codon 816, mostly *KIT* p.D816V. In advanced SM, additional variants in other critical genes encoding transcription factors, signaling molecules, epigenetic regulators and splicing factors, may contribute to disease evolution and the aggressiveness of SM. Based on such additional somatic mutations, clinical variables and the WHO classification, new prognostic scores have been developed, which may help improve prognostication and management of individual patients with SM.

## Figures and Tables

**Table 1:** WHO classification of mastocytosis *.

**Cutaneous mastocytosis (CM)**
maculopapular cutaneous mastocytosis (MPCM) = urticaria pigmentosa (UP)
diffuse cutaneous mastocytosis (DCM)
Mastocytoma of skin (cutaneous mastocytoma)
**Systemic mastocytosis (SM)**
Indolent systemic mastocytosis (ISM)
Smoldering systemic mastocytosis (SSM)
Systemic mastocytosis with associated hematologic neoplasm (SM-AHN)
Aggressive Systemic mastocytosis (ASM)
Mast cell leukemia (MCL)
**Mast cell sarcoma (MCS)**

* The WHO classification of mastocytosis was updated in 2016 [9,10,11].

**Table 2:** Summary of the published data on *IL4R* and *IL13* genes polymorphisms in mastocytosis.

Gene	Polymorhism	Effect on Gene/Protein Function	Effects on Risk of Mastocytosis and Prognosis	References
***IL4R***	p.Q576R (rs 1801275)	gain-of-function	Association p.Q576R of *IL4R* gene with CM and more favorable prognosis	[26]
***IL13***	−1112C > T (rs 18 00925)	−1112T high transcription variant	Presence of −1112T variant increase the risk of SM and correlate with increased tryptase level	[27]

**Table 3:** Summary of the published data on *IL31* gene polymorphisms in mastocytosis [28].

Gene	Polymorhism	Effect on Gene/Protein Function	Effects on Risk of Mastocytosis and Prognosis
***IL31***	−1066G > A (rs 11608363)	Not known	Not increase mastocytosis risk
	−2057G > A (rs 6489188)	Not known	−2057AA genotype increase the risk of mastocytosis in adult patients with CM and SM
	IVS2 + 12A > G	Noncoding intronic variant	AA and AG genotypes increased the risk of mastocytosis in adults and children with CM

**Table 4:** Summary of the published data on *IL6* and *IL6R* gene polymorphisms in mastocytosis.

Gene	Polymorhism	Effect on Gene/Protein Function	Effects on Risk of Mastocytosis and Prognosis	References
***IL6***	−174G > C (rs 1800795)	−174G high transcription variant	No association with mastocytosis	[29]
***IL6R***	p.D358A (rs 9192284)	Misense variant	homozygous AA carriers of the missense variant had lower risk for mastocytosis than those with the AC or CC genotypes	[29]

**Table 5:** Summary of the published data on *TLR* genes polymorphisms in mastocytosis.

Gene	Polymorphism	Effect on Gene/Protein Function	Effects on Risk of Mastocytosis and Prognosis	References
***TLR2***	p.R753Q (rs5743708)	753Q variant encode non-functional receptor	Presence of 753Q variant of TLR-2 gene increased the risk of systemic mastocytosis	[30]
***TLR4***	896A > G (rs496790)	intronic variant	Not increase mastocytosis risk	[30]
***TLR9***	−1237C > T (rs5743836)	CC lower transcription rate	No association with mastocytosis	[30]

## Abbreviations

ADGRE2	Adhesion G protein–coupled receptor E2
ASM	Aggressive Systemic mastocytosis
ASXL1	ASXL Transcriptional Regulator 1
BST	basal serum tryptase
CBL	Cbl Proto-Oncogene
CM	Cutaneous mastocytosis
DNMT3A	DNA Methyltransferase 3 Alpha
ECD	Extracellular domain of KIT
EZH2	Enhancer Of Zeste 2 Polycomb Repressive Complex 2 Subunit
HSC	hematopoietic stem cells
HαT	hereditary alpha-tryptasemia
HVA	Hymenoptera venom allergy
ISM	Indolent systemic mastocytosis
JMD:	Juxtamembrane domain of KIT
KID	Kinase insert domain of KIT
KIT	KIT Proto-Oncogene, Receptor Tyrosine Kinase
MCL	Mast cell leukemia
MC	Mast cells
PTD	phosphotransferase domain of KIT
SM	systemic mastocytosis
SM-AHN	Systemic mastocytosis with associated hematologic neoplasm
MCS	Mast cell sarcoma
*TPSAB1*	Genes encoding α- and β-tryptases
MCAS	MC activation syndrome
MRGPRX2	Mas-related G protein-coupled receptor
NRAS	NRAS Proto-Oncogene, GTPase
PLCG2	Phospholipase Cγ_2_
RUNX1	RUNX Family Transcription Factor 1
SCF	Stem cell factor
SF3B1	Splicing Factor 3b Subunit 1
SRSF2	Serine And Arginine Rich Splicing Factor 2
TET2	Tet Methylcytosine Dioxygenase 2
TLR	Toll-like receptor
U2AF1	U2 Small Nuclear RNA Auxiliary Factor 1
WDSM	well-differentiated systemic mastocytosis
